# Asymptomatic Presentation of Aggressive Ossifying Fibroma:A Case Report

**DOI:** 10.1155/2011/523751

**Published:** 2011-06-30

**Authors:** Roopashri Rajesh Kashyap, Gopakumar R. Nair, Subhas Babu Gogineni

**Affiliations:** ^1^Department of Oral Medicine and Radiology, A.J. Institute of Dental Sciences, Mangalore 575004, Karnataka, India; ^2^Department of Oral Medicine and Radiology, Mahathma Gandhi Dental College & Hospital, Jaipur 302022, Rajasthan, India; ^3^Department of Oral Medicine and Radiology, A.B. Shetty Memorial Institute of Dental Sciences, Mangalore 575018, Karnataka, India

## Abstract

Ossifying fibromas form a part of the spectrum of fibro-osseous lesions of the jaws. They are rare, benign, nonaggressive tumors that are commonly seen in head and neck region. This paper presents the case of a 40-year-old female patient presented with minimal clinical symptoms, diagnosed to be suffering from aggressive form of ossifying fibroma of maxilla involving the maxillary sinus and ethmoid sinus. This paper emphasizes the importance of computed tomography in diagnosing such unapparent aggressive tumors.

## 1. Introduction

Fibro-osseous lesions are a diverse group of processes that are characterized by replacement of normal bone by fibrous tissue containing a newly formed mineralized product [1]. Ossifying fibroma is a rare, destructive, deforming, slow growing, benign fibro-osseous tumor. It is usually found in the craniofacial bones, with the mandible being the most common site. Less commonly, the orbit, paranasal sinuses, or maxilla have also been involved. Computed tomography (CT) imaging plays a major role in detecting the extent of such lesions, their diagnosis, and planning the management. We report a case of an aggressive form of ossifying fibroma of the maxilla that presented with minimal clinical symptoms.

## 2. Case Report

A 40-year-old female patient reported to our department with the complaint of mild fullness over the right cheek area of 7 months duration. It was nonprogressive and asymptomatic. General physical examination did not reveal any abnormalities. Extraorally, lesion presented as fullness with ill-defined borders on right lower 2/3rd of the face. It was approximately of 1 × 1 inch size. Overlying skin was normal in appearance. Palpation did not reveal any abnormality.

**Figure 1:**
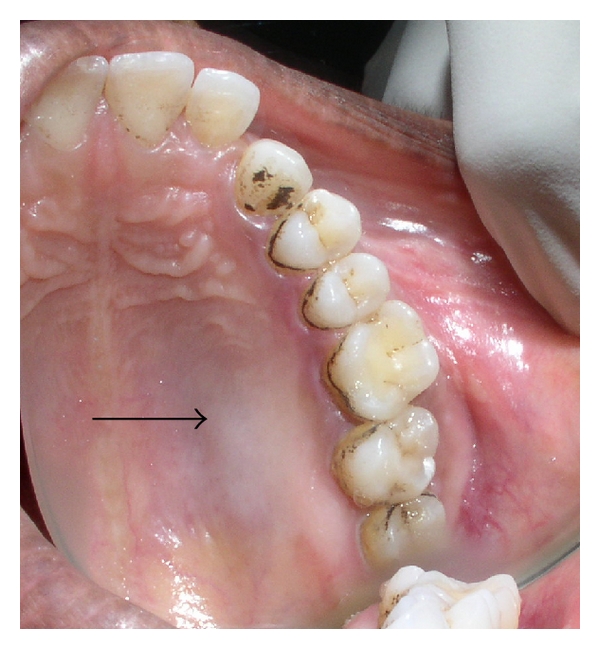
Partial obliteration of buccal vestibule and blanched palatal mucosa.

Intraoral examination revealed diffuse expansion of jaw on right maxillary posterior area, extending anteroposteriorly from distal of upper right second premolar to tuberosity region, measuring approximately 2.5 cm in size. Buccally, the ill-defined enlargement involved only the basal bone, causing partial obliteration of the buccal vestibule. Overlying mucosa was normal in appearance. Palatal expansion was minimal with mild blanching (Figure 1). It was nontender and hard in consistency except at buccal aspect of upper right second molar; where it was soft and suggestive of windowing of bone. Upper right second molar exhibited Grade II mobility. She had good oral hygiene status with no clinically detectable dental caries. Right upper molars were nonresponsive to pulp vitality test and fine needle aspiration was negative. Clinical features were suggestive of a benign neoplasm arising from maxillary basal bone which has windowed the bone in relation to upper right second molar and extended superiorly. A differential diagnosis of benign odontogenic, nonodontogenic, and maxillary antral tumors was considered.

**Figure 2:**
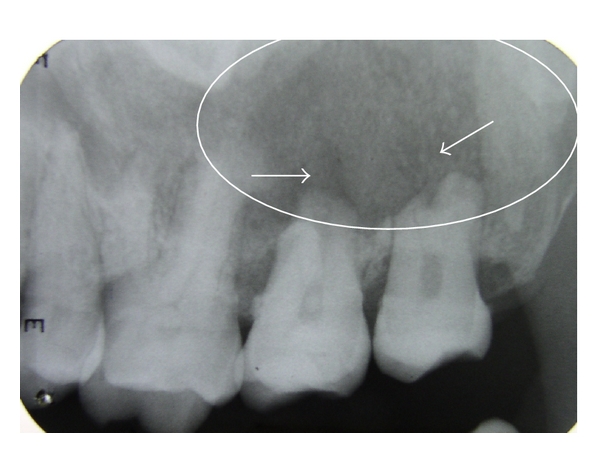
Ill-defined hazy radiolucency with multiple calcified specks and resorption of roots of 17, 18.

On carrying out further investigations, the haematological values were within the normal limits. Rhinoscopy revealed a mass from the middle meatus almost occluding the right nostril approaching the nasal septum. Intraoral periapical radiograph revealed diffuse hazy radiolucency extending from right upper second premolar to third molar causing resorption of the molar roots (Figure 2). Multiple flecks of fine calcifications were noted. Panoramic view additionally revealed haziness of the right maxillary sinus. Paranasal sinus view showed nonuniform opacification of right maxillary sinus with intact orbital margin. Other walls of the sinus are not well defined in this radiograph. As the exact extension of lesion could not be made out, CT imaging was planned. 5 mm cuts were taken through posterior fossa, and 10 mm cuts were taken through supratentorium.

**Figure 3:**
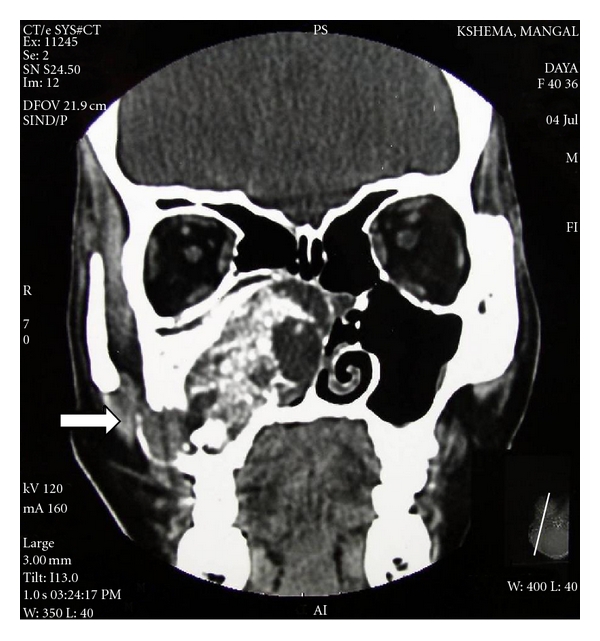
CT-coronal section.

**Figure 4:**
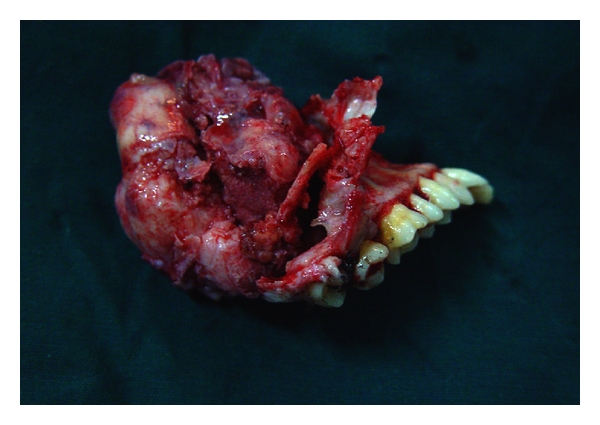
Excised lesion after hemimaxillectomy.

CT sections showed an ill-defined expansile mass lesion completely occluding the right maxillary sinus extending inferiorly upto the right maxillary alveolus (Figure 3). Size of the lesion was approximately 5.6 × 3.3 × 4.2 cm. There was evidence of destruction of medial, anterior, lateral wall of the right maxillary sinus, maxillary alveolus, middle and inferior turbinates. Occlusion of the middle and posterior ethmoidal air sinuses was noticed. Cystic spaces and calcific specks were also noted with part of the lesion showing ground glass appearance. Hence a diagnosis of aggressive ossifying fibroma was suggested. Incisional biopsy followed by excision of the lesion with hemimaxillectomy was carried out (Figure 4). 

**Figure 5:**
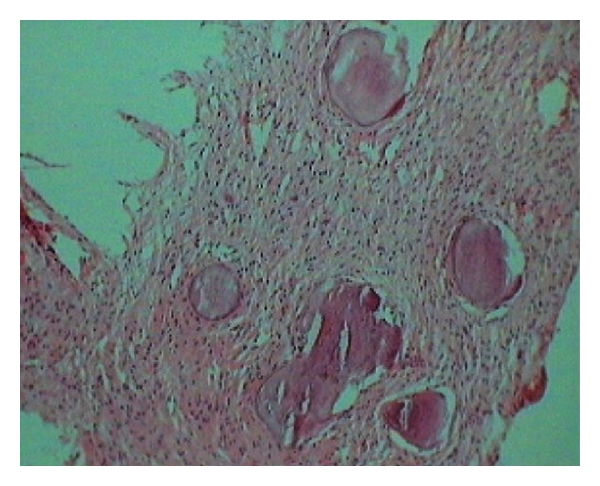
Sections show fibrocellular connective tissue with interspersed spherical calcifications. Most of the areas are highly fibrous with stellate-shaped fibroblasts and plump fibroblasts surrounding the ossifications (H and E, 10x magnification).

**Figure 6:**
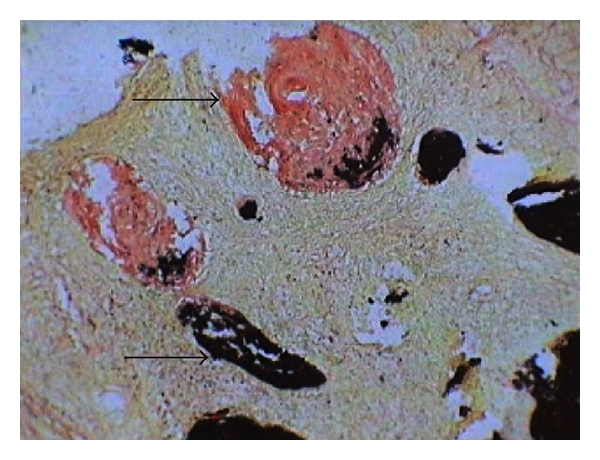
Von Kossa's staining revealed abnormal deposits of calcium and osteoid (10x magnification).

Hematoxylin- and eosin-stained sections showed fibrocellular connective tissue with the spherical cementum like calcifications and ossifications in the form of trabeculae. At few areas, spherical cementum-like calcifications were fused to form globular trabeculae. Most of the areas were highly fibrous with stellate-shaped fibroblasts. Few areas were highly cellular with plump fibroblasts surrounding the ossifications. Trabecular ossifications showed osteoblastic rimming, osteoclasts at few areas, and reversal lines at few sites (Figure 5) Von Kossa's staining revealed abnormal deposits of osteoid (pink) and calcium (black) (Figure 6). Histopathological features of hypercellularity with relatively lesser calcification confirmed the diagnosis of aggressive ossifying fibroma. The patient was rehabilitated with a maxillary prosthesis and was advised for regular followup.

## 3. Discussion

Ossifying fibroma was first described by Menzel in 1872. It is a rare, benign primary bone tumour that occurs most commonly in the jaw. Montgomery in1927 coined the term “ossifying fibroma” [2]. It is a well-demarcated and occasionally encapsulated lesion consisting of fibrous tissue with varying amounts of mineralized material resembling bone and/or cementum. This uncommon tumour can present a diagnostic dilemma for the clinician and the pathologist, owing to overlapping clinical and histomorphologic features. Ossifying fibroma generally manifests in the third or fourth decades of life with a female predilection. Most common site is mandibular premolar-molar region, and about 30% of cases occur in maxilla. When this tumour arises in children, it has been named the *juvenile aggressive ossifying fibroma*, which presents at an earlier age and is more aggressive clinically and more vascular on pathologic exam [3, 4]. Histogenesis of ossifying processes appears to be of two possible origins: the excessive proliferation of periodontal ligaments and a metaplastic process occurring in the connective tissue fibers (nonperiodontal in origin), with the former being more common [4].

Central ossifying fibromas are slow growing and asymptomatic until they cause expansion. One remarkable finding is the large size of the maxillary tumours at the time of diagnosis, probably attributable to the large amount of available space in the maxillary sinus into which they could expand similar to our case. Though the growth was sufficiently large, patient did not present with significant clinical symptoms as it was extending over the sinus region [5]. They are generally encapsulated—a fact that serves to distinguish it from fibrous dysplasia, which may exhibit similar clinicopathological features [6].

The radiographic features of ossifying fibromas, reported in the literature, vary markedly. The majority of them present as well-defined mixed density lesions with few being radiolucent. The radiological appearance depends upon its maturity. They have radiographically well-defined borders, accompanied by marginal sclerosis and a thin cortex. Loss of lamina dura and root resorption and/or divergence of associated teeth may be noted [7–9]. Aggressive lesions in maxilla tend to have ground glass appearance similar to our case [10].

Histologically, the ossifying fibromas are well circumscribed, occasionally encapsulated, consisting of cellular fibrous tissues and thin isolated trabeculae of bones. The bone may show osteoblastic rimming and spherical deposits of calcified material, which are relatively acellular resembling cementum. The lack of consistent osteoblastic rimming of the bone trabeculae in fibrous dysplasia is used to distinguish it from an ossifying fibroma, which is more commonly rimmed by plump osteoblasts [6]. Most authors consider fibrous dysplasia and ossifying fibroma to be histologically similar—with the sole differentiating feature being a fibrous capsule surrounding the latter and infrequently observed in the case of fibrous dysplasia. However, aggressive form of ossifying fibroma may lose its fibrous capsule.

If the lesions are small, they are treated by enucleation. However, larger lesions require radical resection. Recurrence rates of these aggressive forms of ossifying fibromas are about 30% to 38% [11]. Thus a regular followup is necessary.

## 4. Conclusion

The ossifying fibroma of the maxilla is an uncommon benign tumour. Cosmetic and dental occlusal problems are often the first manifestations of these lesions as they are clinically asymptomatic. CT imaging plays a major role in determining the extent of such lesions, their diagnosis, and treatment planning.

## References

[1] Neville BW, Damm DD, Allen CM, Bouquot JE (2002). *Oral and Maxillofacial Pathology*.

[2] Binatli Ö, Erşahin Y, Coşkun S, Bayol Ü (1995). Ossifying fibroma of the occipital bone. *Clinical Neurology and Neurosurgery*.

[3] Mintz S, Velez I (2007). Central ossifying fibroma: an analysis of 20 cases and review of the literature. *Quintessence International*.

[4] Ono A, Tsukamoto G, Nagatsuka H (2007). An immunohistochemical evaluation of BMP-2, -4, osteopontin, osteocalcin and PCNA between ossifying fibromas of the jaws and peripheral cemento-ossifying fibromas on the gingiva. *Oral Oncology*.

[5] Kuta AJ, Worley CM, Kaugars GE (1995). Central cementoossifying fibroma of the maxillary sinus: a review of six cases. *American Journal of Neuroradiology*.

[6] Chong VFH, Tan LHC (1997). Maxillary sinus ossifying fibroma. *American Journal of Otolaryngology*.

[7] MacDonald-Jankowski DS (1998). Cemento-ossifying fibromas in the jaws of Hong Kong Chinese. *Dentomaxillofacial Radiology*.

[8] Speight PM, Carlos R (2006). Maxillofacial fibro-osseous lesions. *Current Diagnostic Pathology*.

[9] Waldron CA (1993). Fibro-osseous lesions of the jaws. *Journal of Oral and Maxillofacial Surgery*.

[10] Langlais RP, Langland OE, Nortje CJ (1995). *Diagnostic Imaging of the Jaws*.

[11] Gunaseelan R, Anantanarayanan P, Ravindramohan E, Ranganathan K (2007). Large cemento-ossifying fibroma of the maxilla causing proptosis: a case report. *Oral Surgery, Oral Medicine, Oral Pathology, Oral Radiology and Endodontology*.

